# Loss of Mef2D function enhances TLR induced IL-10 production in macrophages

**DOI:** 10.1042/BSR20201859

**Published:** 2020-08-21

**Authors:** Michael J. Pattison, Rangeetha Jayaprakash Naik, Kathleen M.S.E. Reyskens, J. Simon C. Arthur

**Affiliations:** Division of Cell Signalling and Immunology, Wellcome Trust Building, School of Life Sciences, University of Dundee, Dow St, Dundee, DD5 1EH, U.K.

**Keywords:** intracellular signaling, macrophages, mef2D, toll-like receptors

## Abstract

Mef2 transcription factors comprise a family of four different isoforms that regulate a number of processes including neuronal and muscle development. While roles for Mef2C and Mef2D have been described in B-cell development their role in immunity has not been extensively studied. In innate immune cells such as macrophages, TLRs drive the production of both pro- and anti-inflammatory cytokines. IL-10 is an important anti-inflammatory cytokine produced by macrophages and it establishes an autocrine feedback loop to inhibit pro-inflammatory cytokine production. We show here that macrophages from Mef2D knockout mice have elevated levels of IL-10 mRNA induction compared with wild-type cells following LPS stimulation. The secretion of IL-10 was also higher from Mef2D knockout macrophages and this correlated to a reduction in the secretion of TNF, IL-6 and IL-12p40. The use of an IL-10 neutralising antibody showed that this reduction in pro-inflammatory cytokine production in the Mef2D knockouts was IL-10 dependent. As the IL-10 promoter has previously been reported to contain a potential binding site for Mef2D, it is possible that the binding of other Mef2 isoforms in the absence of Mef2D may result in a higher activation of the IL-10 gene. Further studies with compound Mef2 isoforms would be required to address this. We also show that Mef2D is highly expressed in the thymus, but that loss of Mef2D does not affect thymic T-cell development or the production of IFNγ from CD8 T cells.

## Introduction

Toll-like receptors (TLRs) are an important group of receptors that allow the innate immune system to recognise invading pathogens. TLRs bind to pathogen associated molecular patterns (PAMPs), typically nucleic acids or cell wall components of the pathogen. TLRs are expressed on a range of immune cell types, including innate immune cells, such as macrophages and dendritic cells. Once TLRs bind their appropriate PAMP, they activate the immune cell and stimulate cytokine production via a core set of signalling pathways. All TLRs, with the exception of TLR3, activate intracellular signalling via the Myd88 adaptor protein which couples TLRs to the MAPK and IKK–NFκB signalling pathways. TLR3 acts via the Trif adaptor and stimulates Tbk1–IRF3 signalling in addition to MAPKs and NFκB, while TLR4 can act via both Myd88 and Trif (reviewed in [1–4]). In macrophages, a major output from TLR signalling is the production of pro-inflammatory cytokines such as TNF, IL-6 and IL-12. In addition to their pro-inflammatory effects, TLRs can also activate anti-inflammatory pathways that are required to prevent excessive inflammation that may be deleterious to the host. These mechanisms include both negative feedback loops in intracellular signalling and also the production of anti-inflammatory cytokines, of which IL-10 is an example [5–7]. IL-10, which is produced by macrophages, dendritic cells and subsets of B cells in response to TLR agonists, is able to inhibit pro-inflammatory cytokine production by macrophages [8,9]. The importance of this feedback loop is demonstrated by the finding that mice with a conditional deletion of IL-10 in macrophages are sensitised to endotoxic shock driven by the TLR4 agonist LPS [10] while total loss of IL-10 function results in sensitisation to colitis in both mice [11] and people [12].

Much of the research on how TLRs induce cytokine production has centred on roles for NFκB, IRF and MAPK signalling [1–4]. The transcription factors that act downstream of MAPKs in these pathways however are not fully elucidated. Mef2 transcription factors comprise a group of 4 proteins, Mef2A, B, C and D, in mammalian cells which have been identified as potential targets of multiple MAPKs [13]. For example, p38 has been shown to phosphorylate and activate Mef2A and C [14–16] while ERK5 phosphorylates and activates Mef2A, C and D [17–19]. They comprise of an N terminal MADS box and MEF domain which are required for DNA binding and dimerization, and a C-terminal TAD (transcriptional activation domain) [13,20]. In mammalian cells, Mef2 form either homodimers or heterodimers with a 2^nd^ Mef2 isoform and the resulting dimers bind to a (C/T)TA(A/T)_4_TA(A/G) consensus sequence in DNA [13,21].

Mef2 transcription factors have well documented roles in development, particularly in muscle, cardiovascular, bone and neuronal development [13,22–25]. Mouse knockout of Mef2C results in embryonic lethality, in part due to defects in cardiovascular development [25]. Mef2A knockout results in perinatal lethality due to cardiovascular defects [26] while Mef2D knockout mice are viable with no overt development phenotype with the exception of a defect in photoreceptor cell development in the eye [27–29]. The ability of Mef2 to act as heterodimers and the similar consensus sequence recognised by the different isoforms suggests that the different isoforms will have overlapping functions. In line with this, compound knockouts have revealed more severe phenotypes in several systems including neuronal development and skeletal muscle regeneration [22,30]. The role of Mef2 in innate immune responses has not been extensively studied. Loss of Mef2C function early in hematopoietic development causes a block at the common lymphoid progenitors (CLP) stage, resulting in greatly reduced numbers of T, B and NK cells but increased numbers of myeloid cells [31]. Deletion of Mef2C specifically in B cells using CD19-Cre did not impair B-cell development indicating that once past the CLP stage of development Mef2C is not essential for B cell development. Mef2C was however required for BCR induced B-cell proliferation and T-cell dependent antibody responses [32,33]. In contrast with BCR regulated responses, Mef2C was not required for TLR or CD40 induced B-cell proliferation [33]. Compensation between Mef2C and Mef2D occurs in B-cell development as deletion of both isoforms together results in a block in B-cell development [34].

With the exception of B cells, the specific roles of Mef2D have not been characterized in detail within other cells of the immune system. We show here that loss of Mef2D does not have a major impact on macrophage, T- or B-cell development. Macrophages from Mef2D knock out mice however showed a decrease in pro-inflammatory cytokine production. This was due to an increased production of IL-10.

## Methods

### Generation of Mef2D knockout mice

**Figure 1:**
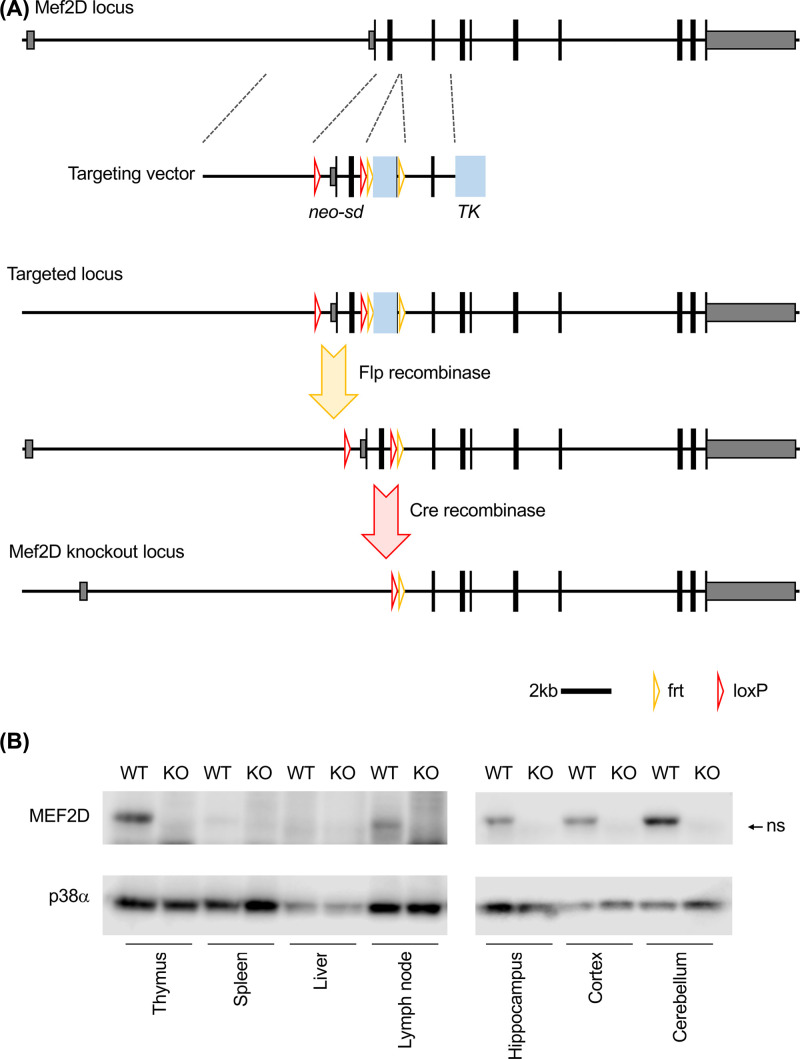
Generation of Mef2D knockout mice The Mef2d knockout was generated using the strategy shown in panel (**A**). A targeting vector was used to insert loxP sites upstream of Mef2D and in intron 2 via homologous recombination in ES cells. Targeted ES cells were used to generate mice carrying the targeted locus, which were then crossed to mice expressing Flpe to remove the neomycin resistance cassette and then Cre to delete exons 1 and 2 in the Mef2D gene, as described in the ´Materials and Methods’. To confirm the knockout was successful, protein extracts from the thymus, spleen, liver, lymph node, hippocampus, cortex and cerebellum of wild type and Mef2D knockout mice were immunoblotted for Mef2D (**B**). Levels of p38α MAPK were also determined to confirm equal loading of sample between the wild-type and Mef2D knockout lysates. ns indicates a non-specific band.

To generate Mef2D knockout mice, a targeting vector was constructed as shown in the schematic in Figure 1. To generate the vector, the arms of homology were PCR amplified from a BAC containing the Mef2D gene and sequenced to ensure they contained no PCR generated mutations. Fragments were then assembled by restriction enzyme cloning into the final vector. A neomycin resistant cassette lacking a polyadenylation sequence [35] but linked to the splice donor site from exon3 of the Mef2D gene was included to allow positive selection in ES cells and a thymidine kinase cassette added for negative selection. The vector was introduced into mouse E14 ES cells by electroporation and ES cell colonies isolated as described [36]. Targeted ES cells were identified by RT-PCR for the neomycin resistant splices onto the 3′ region of Mef2D. ES cells were used to generate chimeric mice via blastocyst injection. Surgery to implant blastocysts in pseudo-pregnant females was carried out under aesthesia with a mix of ketamine and xylazine. Germline transmission was identified by coat colour and confirmed by PCR on ear biopsies. To remove the neomycin resistance gene mice were crossed to Flpe transgenic mice. To generate the total knockout, mice were crossed to a Bal1-Cre (Tg(Nes-cre)^1Wme^, [37]) that resulted in Mef2D deletion in the germline. The resulting heterozygous mice were backcrossed onto C5Bl/6 mice for 12 generations. Routine genotyping of the mice was carried out by PCR of ear biopsies used for identification of individual mice using the primers CTCAATATGTTCTCACTTAGGAGCCTC, TCCCCAAATTGCAACAATATTGGCTATAG and AGACCACCTGCCTAAAGTCACATG which, when used together, would give a 304 bp band for a wild-type allele and 240 bp band for a knockout allele.

Wild type C57Bl6J mice for backcrossing were obtained from Charles River Laboratories U.K. Animals were maintained under specific pathogen-free conditions in line with EU and U.K. regulations. Nonbreeding mice were housed in same-sex groups, in individually ventilated sterile cages and had free access to water and food (standard diet R&M1 or R&M3 from SDS Special Diets Services). Animals were maintained in rooms with controlled 12-h/12-h light/dark cycle, 21 °C temperature, and relative humidity of 45–65%. Mice were culled using a raising concentration of CO_2_ for 5 min and death confirmed via cervical dislocation. All the work was performed at the University of Dundee under a U.K. Home Office project license (70/7901) in accordance with U.K. and EU regulations and approved by local ethical review by the University of Dundee Ethical Review Committee. Mice were used for isolation of tissue at 10 to 14 weeks of age.

### Flow cytometry

Thymi and spleens were removed and disaggregated in RPMI media. Spleens were treated with RBC lysis buffer (Sigma). Single cell suspensions, diluted in PBS, were used to analyse total cell counts on BD FACSVerse™ (BD Biosciences). For phenotyping, cells were washed with FACS buffer (1% BSA in PBS) and Fc γ receptors were blocked with Mouse BD Fc Block (BD Pharmingen) for 15 min at 4°C. Cell surface markers on thymocytes were then stained with anti-Thy1.2-APC, anti-CD4-PE, anti-CD8-BV421 and anti-TCRβ-FITC in FACS buffer for 20 min at 4°C. T and B cells in the spleen were stained with anti-TCRβ-FITC, anti-CD4-PE, anti-CD8-APC and anti-CD19-PerCP-Cy5.5. Myeloid cells in the spleen were stained with anti-F4/80-BV421, anti-CD11b-PE, anti-CD11c-APC and anti-Gr-1-PerCP-Cy5.5, while FITC was used as dump channel for anti-TCRβ and anti-CD19. Antibodies used were from BD Biosciences or BioLegend. Stained cells were washed and resuspended in FACS buffer and acquired on BD FACSCanto™ II (BD Biosciences) using FACSDiva software. Analysis was done on FlowJo.

### Expansion of CD8 T cells and measurement of T cell IFNγ production

Spleens were removed, disaggregated in RPMI media and treated with RBC lysis buffer (Sigma). Single cell suspensions were adjusted to 2.5 × 10^6^ cells/ml in T-cell media (RPMI-1640 supplemented with 10% heat inactivated FBS, 4 mM L-glutamine, 10 mM HEPES, 100 U/ml penicillin, 100 µg/ml streptomycin, 100 µM non-essential amino acids, 1 mM sodium pyruvate and 50 µM 2-mercaptoethanol). Cells were stimulated with 0.5 µg/ml of soluble anti-CD3 monoclonal antibody (145.2C11) and 20 ng/ml IL-2 for 36 h. Cells were then washed, counted and cultured at 1 × 10^6^ cells/ml in T-cell media containing 20 ng/ml IL-2 every day for 7 days. On day 7, cells were washed and resuspended at 0.5 × 10^6^ cells/ml in T-cell media containing 20 ng/ml IL-2. They were then either left unstimulated or stimulated with anti-CD3 (1 µg/ml) and anti-CD28 (0.5 µg/ml) or Phorbol 12, 13-Dibutyrate (PDBu; 20 ng/ml) and ionomycin (0.5 µg/ml) for 4 h. Levels of interferon γ were measured in the media by ELISA (eBioscience) according to manufacturer’s protocol.

### Culture of bone marrow derived macrophages

Bone marrow derived macrophages (BMDMs) were cultured as described [38]. Briefly, bone marrow was flushed from the femurs of one mouse using PBS. Cells were then pelleted by centrifugation and cultured on bacterial grade plastic for 7 days in BMDM media (DMEM supplemented with 10% heat inactivated fetal bovine serum, 2 mM L-glutamine, 100 units/ml penicillin G, 100 μg/ml streptomycin, 0.25 μg/ml amphotericin and 5 ng/ml recombinant M-CSF). Cells were then detached by scraping in versene (Invitrogen) and re-plated on tissue culture plastic in BMDM media. Cells were stimulated with 100 ng/ml LPS (E. coli O26:B6, Sigma L2654).

### Immunoblotting

For immunoblotting, cells were lysed in 50 mM Tris-HCl (pH 7.5), 1 mM EGTA, 1 mM EDTA, 1 mM sodium orthovanadate, 50 mM sodium fluoride, 1 mM sodium pyrophosphate, 10 mM sodium glycerophosphate, 0.27 M sucrose, 1% (vol/vol) Triton X-100, 0.1% (vol/vol) 2-mercaptoethanol and complete proteinase inhibitor cocktail (Roche). Lysates were clarified by centrifugation (13,000 rpm for 10 min at 4°C) and supernatants snap-frozen and stored at −80°C. Protein concentration was determined with Coomassie Protein Assay Reagent (Thermo Scientific). Proteins were separated on 10% polyacrylamide gels and immunoblotting carried out using standard techniques. Antibodies against Mef2D phospho-Thr180/Tyr182 p38, total p38, phospho-Thr202/Tyr204 ERK1/2, total ERK1/2, phospho-Ser133 CREB, phospho S376 MSK1 and phosphor Ser933 p105 were from Cell Signaling. The antibody raised against total MSK1 was described previously [39]. Secondary antibodies conjugated to horseradish peroxidase and bands visualized via Clarity ECL (Bio-Rad) using either film or a Li-Cor Odyssey Fc scanner.

### qPCR

Total RNA was prepared from cells using Qiagen RNeasy kits in accordance with the manufacturer’s protocol. RNA was reverse transcribed using iScript (Bio-Rad). qPCR was carried out using SYBR green based reagents (Takara). mRNA induction was determined relative to unstimulated wild-type cells as described [40,41]. GAPDH and/or 18S levels were used to normalise for differences in total RNA concentrations. Primer sequences have been described previously [40,41].

### Determination of cytokine levels

IL-10, TNF, IL-6 and IL-12p40 were measured using a Luminex based assay (Bio-plex, Bio-Rad) according to the manufacturer’s protocol.

## Results

### Generation of Mef2D knockouts

To generate the Mef2D knockout, conventional gene targeting techniques were used to place loxP sites upstream of exon 2 and downstream of exon 3, as indicated in the schematic in Figure 1A. Initially heterozygous mice were bred to transgenic mice expressing a Flpe transgene in order to remove the neomycin resistance gene. To generate a total Mef2D knockout, the Mef2D floxed mice were bred to a germline Cre transgenic mouse to delete exons 2 and 3. This removes the first 86 amino acids of Mef2D including the MADS box that is required for dimerization and DNA binding. The resulting heterozygous Mef2D mice were bred away from the Flpe and Cre transgenes and then crossed to generate the homozygous knockout. The homozygous knockout mice were viable and born at close to the expected frequency from heterozygous matings (28%, *n*=54). The mice had no apparent gross abnormalities and were fertile. To confirm the knockout resulted in a loss of Mef2D protein, immunoblotting was used to detect Mef2D in lysates from the thymus, spleen, liver and brain. In wild-type mice levels of Mef2D were highest in the thymus, hippocampus, cortex and cerebellum with lower levels in the lymph nodes and spleen. Mef2d was not detected in the liver. Mef2D was not detected in lysates from the knockout mice (Figure 1B).

### Mef2D is not essential for T-cell development

**Figure 2:**
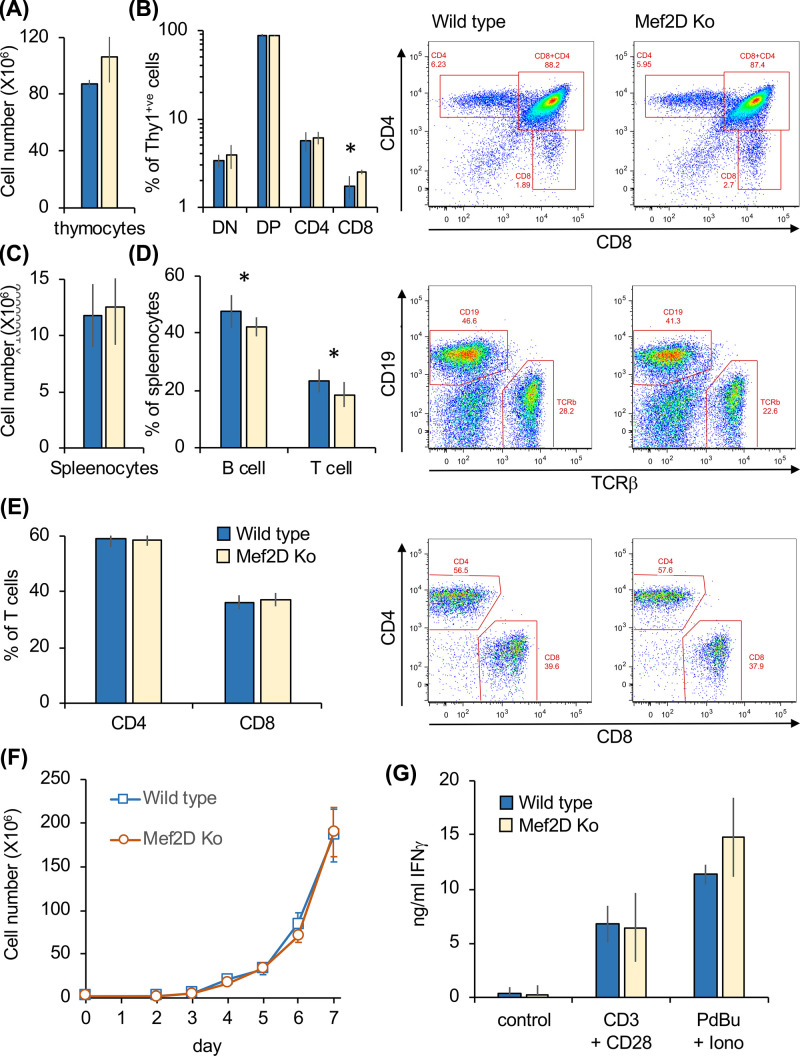
Mef2D is not required for T- or B-cell development Thymi were isolated from wild-type and Mef2D knockout mice. Thymocytes were stained for Thy1, CD4 and CD8 and analysed by flow cytometry. The numbers of thymocytes is shown in panel (**A**). Cells were gated on Thy1 expression and the percentage of CD4 and CD8 positive cells is shown in panel (**B**), with quantification from four wild-type and 5 Mef2D knockout mice shown on the left and representative plots on the right. In panels (**C–E**), spleens were isolated from nine wild-type and nine Mef2D knockout mice and red blood cells lysed as described in the ‘Materials and Methods’ section and then cells stained for CD19, TCRβ, CD4 and CD8. Number of splenocytes is shown in panel (**C**). The percentage of T cells and B cells in the spleen is shown in panel (**D**). The percentage of CD4 and CD8 cells in the T cell (TCRβ+ve) cells is shown in panel (**E**). In panels (**F** and **G**), CD8 T cells were expanded from splenocytes *ex vivo* as described in the ‘Materials and Methods’ section. The number of cells present over time in cultures from wild-type and Mef2D knockout mice is shown in (**F**). On day 7, cells were either left unstimulated or stimulated with either anti-CD3 (1 µg/ml) and anti-CD28 (0.5 µg/ml) or PdBu (20 ng/ml) and ionomycin (0.5 µg/ml) for 4 h. Levels of interferon γ secreted into the media were measured by ELISA panel (**G**). For panels (**F** and** G**), data show mean and standard deviation of cultures from 4 mice per genotype. A *P* value of less than 0.05 (two-tailed Student’s *t-*test) is indicated by *.

As Mef2D was high in the thymus, we examined whether the loss of Mef2D had a major impact on T-cell development. Thymi and spleens were isolated and immune cell subsets determined by flow cytometry. Cell number in the thymus was similar between wild-type and Mef2D knockout animals (Figure 2A). In the thymus T cells pass through several developmental stages, defined by their expression of CD4 and CD8; initially T cells express neither CD4 or CD8 (DN or double negative cells) before up-regulating the expression of both CD4 and CD8 (DP or double positive cells). Finally, the cells lose expression of either CD4 or CD8 to give single positive (SP) cells which then exit the thymus [42,43]. The percentage of DN, DP and CD4 SP cells was similar between wild-type and Mef2D knockouts, although there was a minor increase in the percentage of CD8 SP cells in the Mef2D knockout (Figure 2B). Analysis of splenocytes following red blood cell lysis showed similar numbers of cells in the Mef2D knockout and wild-type mice spleens (Figure 2C). Staining for CD19 (B cells) and TCR (T cells) showed that the percentages of both T and B cells was slightly lower in the Mef2D knocklout mice (Figure 2D). When corrected for the total cell number, this difference was maintained (4.63 × 10^7^ and 1.94 × 10^7^ for T and B cells respectively in wild-type spleens vs 3.3 × 10^7^ and 1.48 × 10^7^ in Mef2D knockouts); however, the difference was no longer significant (*P*>0.05, Student’s t-test). Analysis of CD4 and CD8 within the T-cell population showed no difference between the ratio of CD4 and CD8 T cells between wild-type and Mef2D knockout spleens (Figure 2E). Stimulation of splenocytes with a combination of anti-CD3 and anti-CD28 to trigger the TCR followed by maintenance in IL-2 resulted in a similar expansion of cytotoxic T cells from the splenocytes of wild-type and Mef2D KO (Figure 2F). On day 7, restimulation with either anti-CD3 and CD-28 or PdBu and ionomycin resulted in similar levels of interferon γ production in wild-type and Mef2D knockout cells (Figure 2G).

### Mef2D regulates IL-10 induction in BMDMs

**Figure 3:**
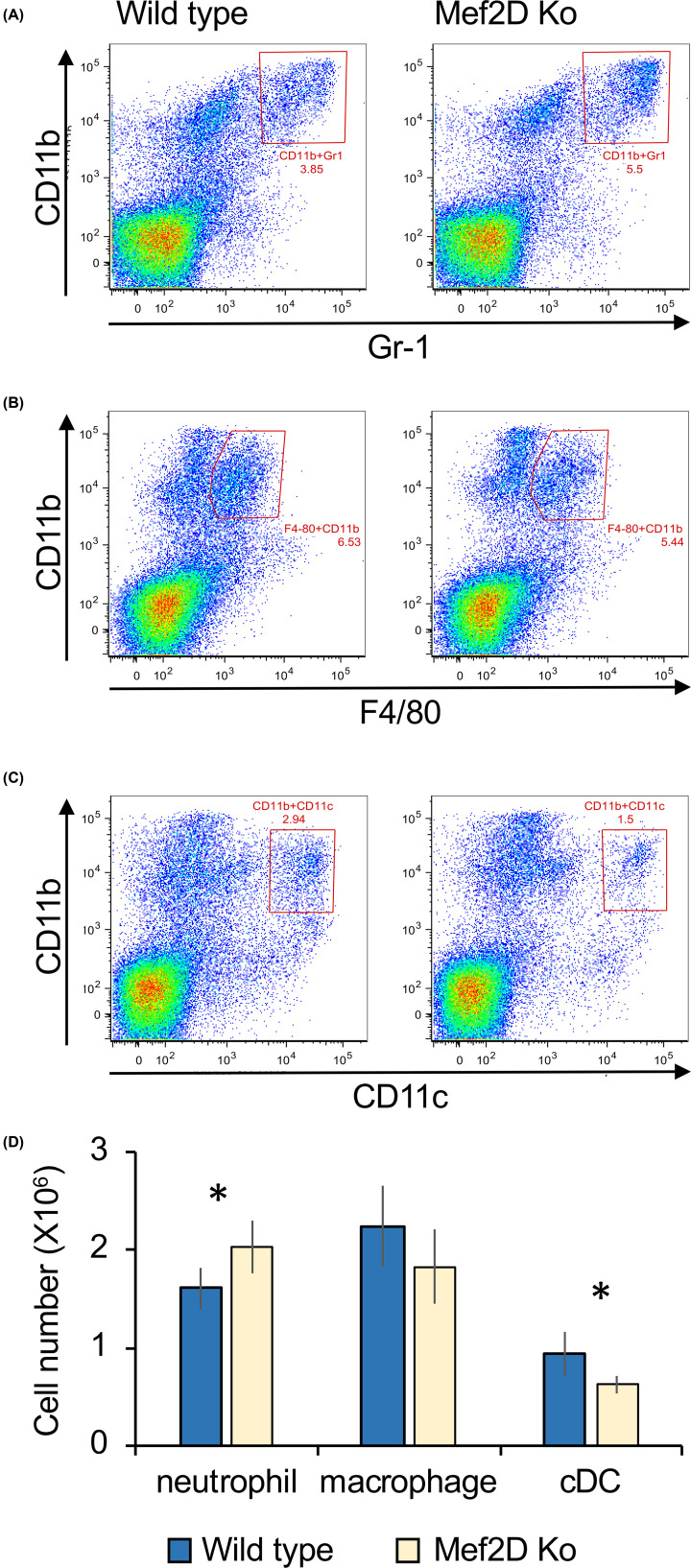
Mef2D is not essential for myeloid cell development Spleens were isolated from five wild-type and four Mef2D knockout mice and red blood cells lysed as described in the ‘Materials and Methods’ section and then cells stained for CD19, TCRβ and the indicated markers. Representative plots to identify CD11b / Gr1 positive neutrophils are shown in panel (**A**), CD11b / F4/80 positive macrophages in panel (**B**) and Cd11b / CD11c positive conventional dendritic cells in panel (**C**). Average cell numbers and standard deviation for the three cell populations are shown in panel (**D**). A *P* value of less than 0.05 (two-tailed Student’s *t-*test) is indicated by *.

Myeloid populations were similar between wild-type and Mef2D knockout spleens, although, Mef2D knockout mice did have a small increase in CD11b/Gr1 positive neutrophils in the spleen (Figure 3A,D). The numbers of CD11b/F4/80 positive macrophages in the spleen were not significantly different between wild-type and Mef2D knockouts (*P*>0.05, Student’s *t-*test, Figure 3B,D). There was a small decrease in the total number of CD11b/CD11c positive cells, which would include CD8^-ve^ classical dendritic cells. (Figure 3C,D). Overall, the above data indicate that loss of Mef2D alone does not have a major impact on immune cell development.

**Figure 4:**
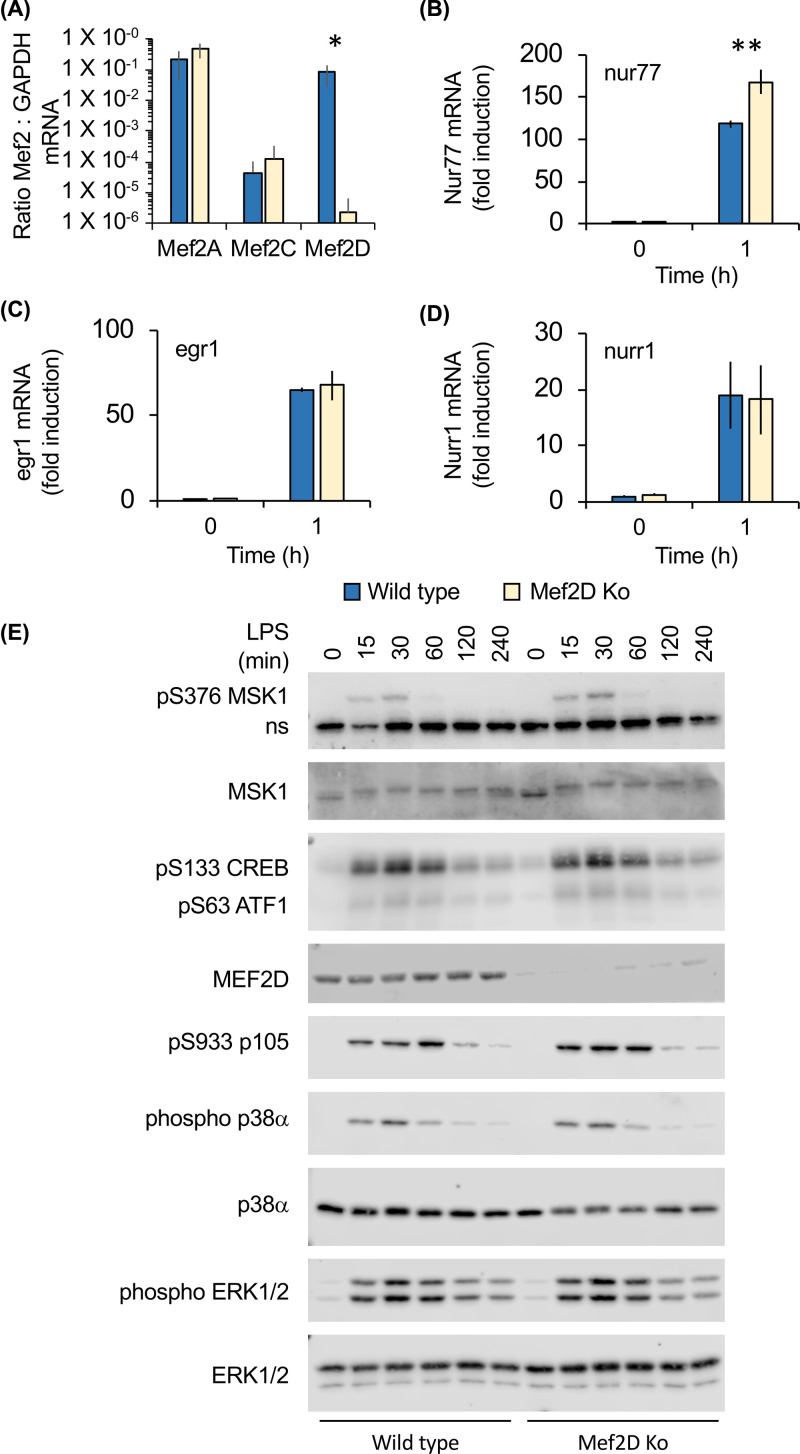
Mef2D knockout does not inhibit TLR4 induced signalling in macrophages (**A**) Bone marrow derived macrophages were isolated from wild-type and Mef2D knockout mice. The levels of Mef2A, Mef2C and Mef2D mRNA relative to GAPDH mRNA were then determined by qPCR. Error bars represent the standard deviation of independent cultures from four mice per genotype. (**B–E**) BMDMs were stimulated with 100 ng/ml LPS for the indicated times. Total RNA was isolated and the levels of nur77 (B), egr1 (C) and nurr1 (D) mRNA determined by qPCR. Fold change is expressed relative to the level in unstimulated wild-type macrophages. Error bars represent standard deviation from independent cultures from three mice per genotype. A *P* value (two-tailed Student’s *t-*test) between wild-type and Mef2D knockout cells of less than 0.001 is indicated by ** and less than 0.05 by *. (**E**) Cells were lysed and samples run on SDS poly-acrylamide gels and blotted for the indicated phospho or total proteins (**E**). ns indicates a non-specific band detected by the phospho MSK1 antibody.

Previous studies have found that Mef2D is expressed in human monocytes and is up-regulated as the cells differentiate into macrophages [44]. We therefore looked at the effect of Mef2D knockout on macrophage function. Loss of Mef2D did not block the M-CSF driven differentiation of bone marrow cell into macrophages *in vitro* (data not shown). Analysis of mRNA levels for the different Mef2 isoforms showed that the wild type BMDMs expressed high levels of both Mef2A and Mef2D and low levels of Mef2C (Figure 4A). Mef2B mRNA was not detected in the samples (data not shown). Mef2 transcription factors have been linked to the regulation of several genes, including nur77 [21,45–48]. In BMDMs, nur77 mRNA was induced by the TLR4 agonist LPS (Figure 4B). Surprisingly, nur77 mRNA induction was slightly increased in Mef2D knockout cells (Figure 4B). In contrast, the induction of two other immediate early genes, egr1 and nurr1, in response to LPS was not affected by Mef2D knockout (Figure 4C,D), indicating that any changes are restricted to a subset of genes and not a global up-regulation in the response. In addition to Mef2, CREB has also been found to regulate the nur77 promoter [41]. Downstream of LPS, CREB is phosphorylated by the kinases MSK1 and 2 on Ser133 [40] and the MSK-dependent phosphorylation of CREB is required for maximal nur77 induction in fibroblasts and BMDMs [49,50]. We therefore looked at the effect of Mef2D knockout on CREB phosphorylation in response to LPS in BMDMs. The activation of MSK1, as judged by its phosphorylation on Ser376 or the phosphorylation-induced band-shift in total MSK1 was also unaffected. In line with this CREB phosphorylation was also unaffected (Figure 4E).

**Figure 5:**
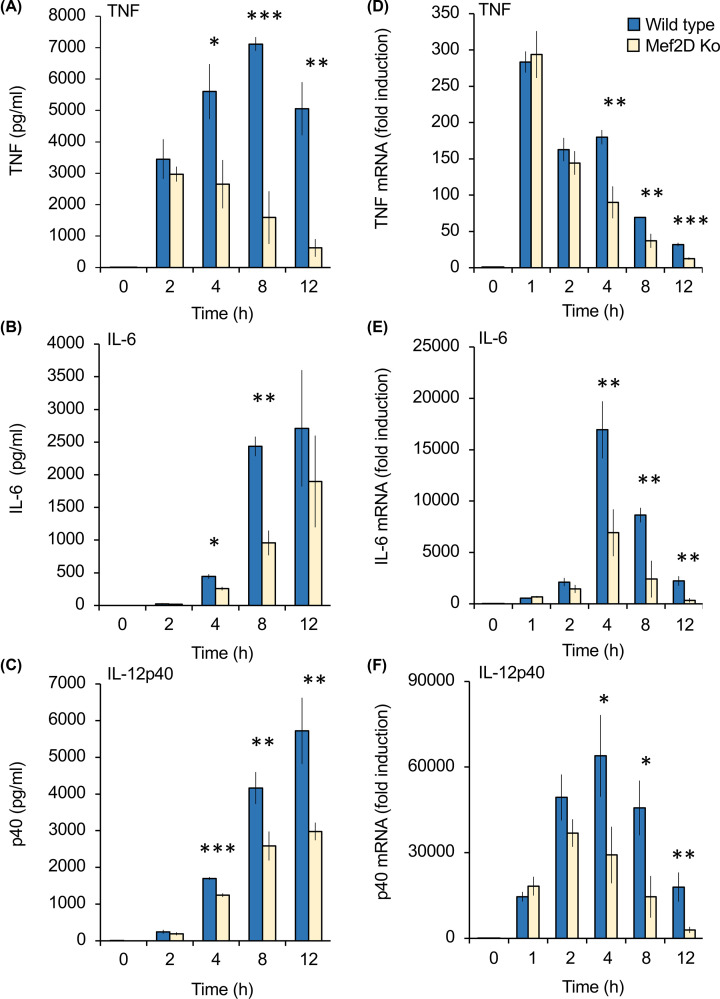
Mef2D knockout results in decreased pro-inflammatory production in LPS stimulated macrophages Bone marrow derived macrophages were isolated from wild-type and Mef2D knockout mice. Cells were stimulated with 100 ng/ml LPS for the indicated times. The levels of TNF (**A**), IL-6 (**B**) and IL-12p40 (**C**) secreted into the media are shown in panel (B). Total RNA was isolated and the levels of TNF (**D**), IL-6 (**E**) and IL-12p40 (**F**) mRNA determined by qPCR. Values are expressed relative to the level in unstimulated wild type macrophages (D–F). Error bars represent standard deviation from independent cultures from three mice per genotype. A *P* value (two tailed Student’s *t-*test) between wild-type and Mef2D knockout cells of less than 0.001 is indicated by ***, less than 0.01 by ** and less than 0.05 by *.

LPS stimulation of BMDMs results in the production of multiple pro-inflammatory cytokines, including TNF, IL-6 and IL-12p40. Following 2 h of stimulation, wild-type and Mef2D BMDMs secreted similar amounts of TNF, however from 4 to 12 h of stimulation Mef2D knockouts secreted less TNF than wild-type cells (Figure 5A). A similar pattern was also seen for IL-6 (Figure 5B) and IL-12p40 (Figure 5C). To determine if the changes in these cytokines was also reflected at an mRNA level, qPCR was used to determine mRNA levels for these cytokines. As seen for secreted protein, little difference between wild-type and Mef2D knockout BMDMs was observed for TNF, IL-6 and IL-12p40 mRNA levels following 1 to 2 h of LPS stimulation (Figure 5D–F). However, from 4 h on lower levels of the mRNA for these cytokines was observed in Mef2D knockout BMDMs relative to wild-type cells, with this effect being more pronounced for IL-6 and IL-12p40 than TNF (Figure 5D–F). The induction of pro-inflammatory cytokines downstream of TLRs requires the activation of NFκB and MAPK signalling. The activation of ERK1/2 and p38α as judged by phosphorylation on their TXY activation motifs was not affected by Mef2D knockout. NFκB is activated by the IKK complex that phosphorylates both IκB and the p105 NFκB subunit. Mef2D knockout did not affect the phosphorylation of p105 downstream of LPS activation, suggesting that NFκB activation was not blocked (Figure 4E).

**Figure 6:**
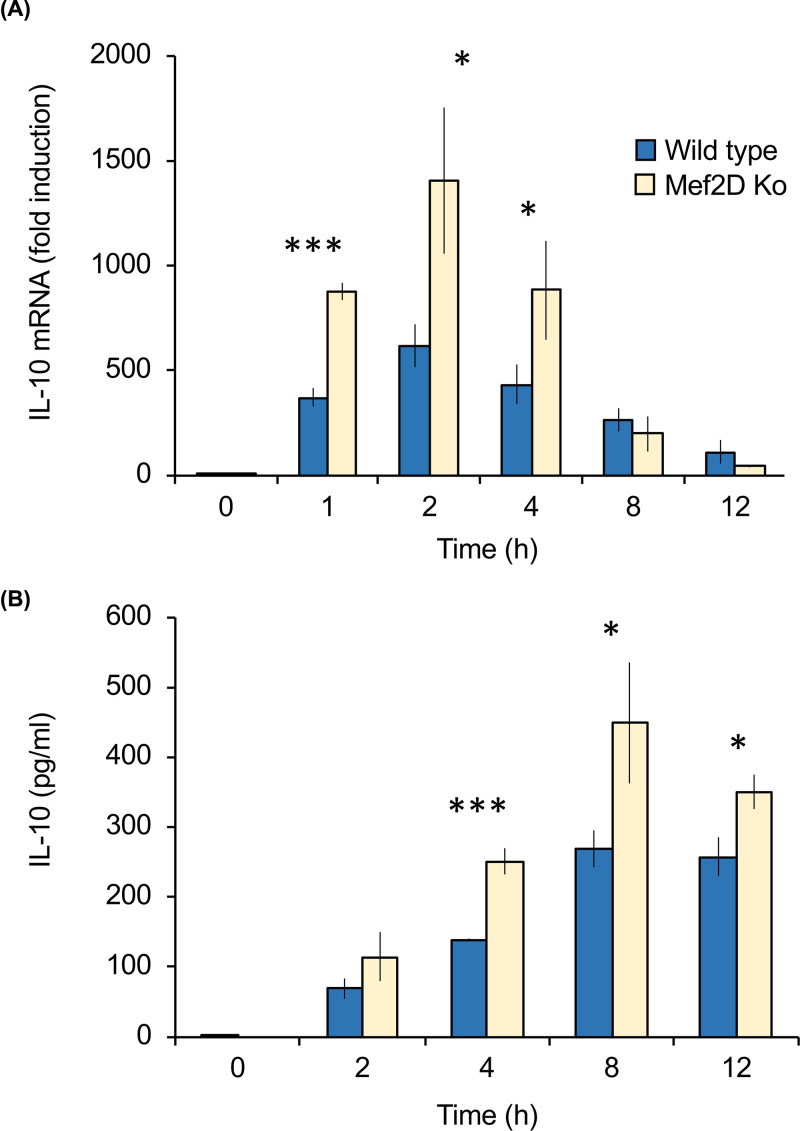
Mef2D acts as a negative regulator of LPS induced IL-10 production Bone marrow derived macrophages were isolated from wild type and Mef2D knockout mice. Cells were stimulated with 100 ng/ml LPS for the indicated times. Total RNA was isolated and the levels of IL-10 mRNA determined by qPCR. Values are expressed relative to the level in unstimulated wild type macrophages (**A**). The levels of IL-10 secreted into the media are shown in (**B**). Error bars represent standard deviation from independent cultures from 3 mice per genotype. A *p* value (2 tailed Students ttest) between wild type and knockout cells of less than 0.001 is indicated by *** and less than 0.05 by *.

In addition to stimulating the production of pro-inflammatory cytokines, LPS also stimulates the production of the anti-inflammatory cytokine IL-10. Similar to nur77, IL-10 is another gene whose transcription has been linked to both CREB and Mef transcription factors, the induction of IL-10 mRNA in response to LPS was determined. Mef2D knockout BMDMs exhibited a higher induction of IL-10 mRNA in response to LPS than wild-type cells (Figure 6A). In line with the increased IL-10 mRNA levels, Mef2D knockout BMDMs also secreted higher levels of IL-10 in response to LPS compared with wild-type cells (Figure 6B).

**Figure 7:**
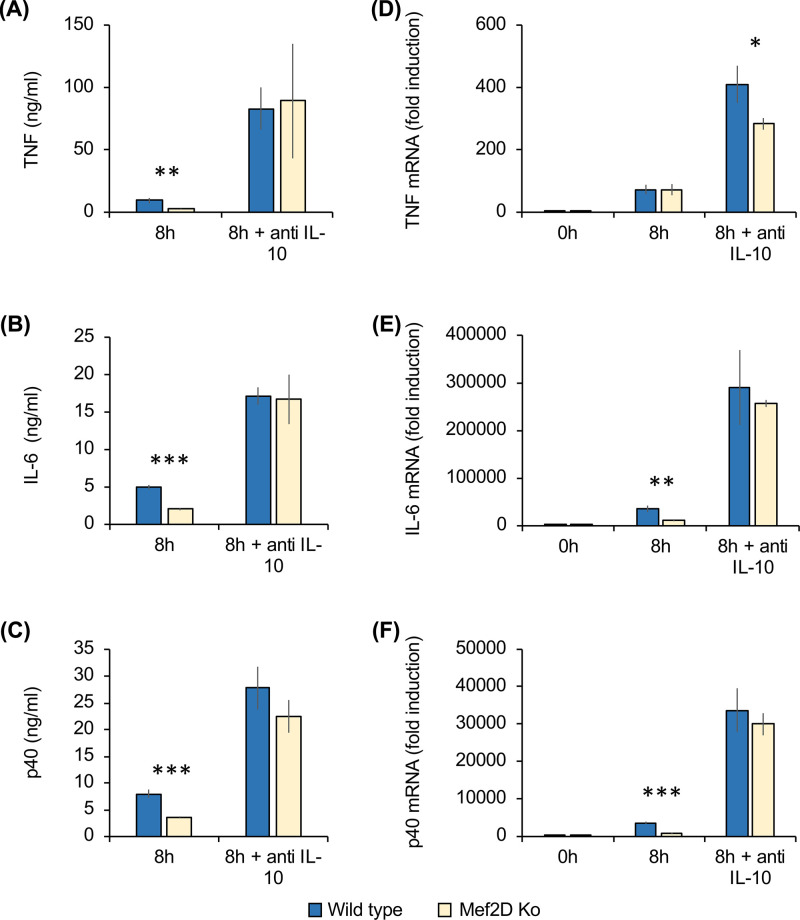
Decreased LPS induced pro-inflammatory cytokine production in Mef2D knockout macrophages is dependent on IL-10 Bone marrow derived macrophages were isolated from wild-type and Mef2D knockout mice. Cells were stimulated with 100 ng/ml LPS for 8 h in the presence or absence of a neutralising antibody against IL-10. The levels of TNF (**A**), IL-6 (**B**) and IL-12p40 (**C**) secreted into the media are shown in panel (B). Total RNA was isolated and the levels of TNF (**D**), IL-6 (**E**) and IL-12p40 (**F**) mRNA determined by qPCR. Values are expressed relative to the level in unstimulated wild-type macrophages (D–F). Error bars represent standard deviation from independent cultures from three mice per genotype. A *P* value (Student’s *t-*test) between wild-type and knockout cells of less than 0.001 is indicated by ***, less than 0.01 by ** and less than 0.05 by *.

IL-10 is able to repress the production of TNF, IL-6 and IL-12p40 in response to LPS [40]. To determine if the decreased production of pro-inflammatory cytokines in the Mef2D knockout BMDMs was due to their increased production of IL-10 (Figure 5), the LPS stimulation was repeated in the presence or absence of an IL-10 neutralising antibody. As observed in the previous experiment, LPS stimulated TNF, IL-6 and IL12p40 secretion in the absence of the IL-10 antibody was lower in Mef2D knockouts relative to wild-type BMDMs (Figure 7A–C). Incubation of the cells with the IL-10 neutralising antibody increased the LPS-stimulated production of TNF, IL-6 and IL-12p40 in both wild-type and Mef2D knockout BMDMs compared to LPS stimulation in the absence of the IL-10 antibody. In the presence of the IL-10 neutralising antibody, however, the levels of TNF, IL-6 and IL-12p40 were similar between the wild-type and Mef2D knockout macrophages (Figure 7A–C). Analysis of mRNA induction showed that in the presence of the neutralising antibody for IL-10, the levels of IL-6 and IL-12p40 mRNA induction were similar between wild-type and Mef2D knockout cells, although a small decrease remained in the levels of TNF mRNA (Figure 7D–F).

## Discussion

In the present study, we used Mef2D knockout mice to examine the potential role of Mef2D in T cells and macrophages. The strategy we used to target Mef2D resulted in the removal of exons 2 and 3, which deleted the coding sequence for amino acids 1 to 86. The next potential in frame ATG is at position 142, meaning any protein would lack dimerization and DNA-binding domains critical for function. Three other Mef2D knockouts have been reported. The first removed exon 3, and the resulting splicing from exon 2 to 4 resulted in an inactive Mef2D fragment lacking amino acids 18 to 86 [27]. The other 2 knockouts removed exons 2 to 4 or 2 to 6 [28,29]. Similar to the knockout reported here, all these lines were viable and fertile. Andzelm et al. and Omori et al. both reported issues with the maintenance of photoreceptor cells in the retina [28,29], while Kim et al. reported protection to cardiac hypertrophy and fibrosis in response to pressure overload and beta-chronic adrenergic stimulation in the heart [27], none of which were addressed in the current study.

Mef2A and Mef2D have previously been reported to be active transcription factors in T cells and have been suggested to regulate nur77 induction downstream of Ca^2+^ signalling [47]. Although Mef2D was strongly expressed in the thymus, its loss did not appear to compromise T-cell development, as judged by normal numbers of T cells in the thymus and spleen (Figure 2). Analysis of data in the ImmGen database confirmed that Mef2D was expressed throughout T cell developmwnt with highest levels seen in DP cells in the thymus and lower levels seen in naive CD4 and CD8 T cells in the spleen (Supplementary Figure S1). Among the other Mef2 isoforms, the expression of Mef2A mirrored that of Mef2D. Mef2C was expressed in DN1 cells but was then down-regulated while Mef2B was only expressed at very low levels. A recent proteomic study of T cells has also confirmed the expression of Mef2A and Mef2D but not Mef2B or Mef2C in peripheral T cells [51]. Mef2A knockout mice exhibit a high degree of postnatal mortality [26], however the effects of Mef2A knockout on T-cell development have not been reported. As discussed further below, it is possible that Mef2A may compensate for the loss of Mef2D and that a double knockout may be required to define the roles of these transcription factors in T cells.

Loss of Mef2D did also not prevent myeloid cell development, as indicated by the presence of neutrophils and macrophages in the spleen. There was a small decrease in CD11b^+ve^/CD11c^+ve^ cells in the spleen, a population that would include the CD8-ve subset of classical dendritic cells. A detailed analysis of myeloid developmental precursors or dendritic cells was not carried out and it is possible that Mef2D knockout would have effects in these processes.

Macrophages were also found to express Mef2A and D, however unlike T cells they also expressed Mef2C. We show here that in bone marrow derived macrophages, loss of Mef2D results in an elevated production of IL-10 in response to LPS compared with wild-type cells. As a result of this increased IL-10 production, Mef2D knockout macrophages secreted lower levels of TNF, IL6 and IL-12p40. Previous studies have identified a potential Mef2-binding site in the IL-10 promoter and shown that overexpression of Mef2D increased IL-10 promoter activity in human T cells [52,53]. shRNA-mediated knockdown of Mef2D in the BV2 microglial cell line also reduced IL-10 induction [54]. In contrast, BMDMs IL-10 production was higher in Mef2D knockouts than wild-type cells (Figure 5). Furthermore, there was a slight increase in the induction of the Mef2 target gene nur77. One potential explanation for this could be compensation between Mef2 isoforms. In addition to Mef2D, bone marrow derived macrophages also express Mef2A and Mef2C. It is possible that the increased transcription of IL-10 in the Mef2D knockout may result from the replacement of Mef2D on the IL-10 promoter with a different Mef2 isoform, which may have a higher ability to activate the IL-10 promoter. Of note, it has been demonstrated that deletion of Mef2D in granule neurons in the cerebellum results in the recruitment of Mef2A to a subset of promoters normally bound by Mef2D [55]. Redundancy between Mef2 isoforms is also established in the literature. Partial redundancy between Mef2C and Mef2D has been reported in B-cell development. Redundancy of Mef2A, Mef2C and Mef2D has been observed in the CNS; brain specific Mef2A/D double knockouts are viable whereas brain specific deletion of Mef2A, C and D results in postnatal mortality and neuronal apoptosis [22]. Combined deletion of Mef2A, C and D is also required to block skeletal muscle regeneration with single knockouts having no impact [30]. A further question is how Mef2 transcription factors are regulated in macrophages. Mef2 isoforms have been reported to be MAPK substrates in other systems [13]. TLRs are known to activate multiple MAPK pathways in macrophages and it would therefore be possible that Mef2 is controlled by MAPKs in response to TLR stimulation; however, further studies would be required to establish this.

In conclusion, we show here that Mef2D plays a role in the regulation of the important anti-inflammatory cytokine IL-10 in macrophages. Further studies will however be required to address functional compensation between Mef2 isoforms in macrophages.

## Supplementary Material

Supplementary Figure S1

## Abbreviations

BMDM: bone marrow derived macrophage
PAMP: pathogen associated molecular pattern
SP: single positive
TLR: Toll-like receptor
