# Supplementary material for: Loss of Mef2D function enhances TLR induced IL-10 production in macrophages
Source: Biosci Rep. 2020 Aug 21;40(8):BSR20201859. doi: 10.1042/BSR20201859 (PMC7442974; doi:10.1042/BSR20201859)
Supplement: Supplementary Figure S1 [file BSR-2020-1859_supp.pdf]

Loss of Mef2D function enhances TLR induced IL-10 production in macrophages.

Michael J. Pattison, Rangeetha Jayaprakash Naik, Kathleen M. S. E.Reyskens and J. Simon C. Arthur

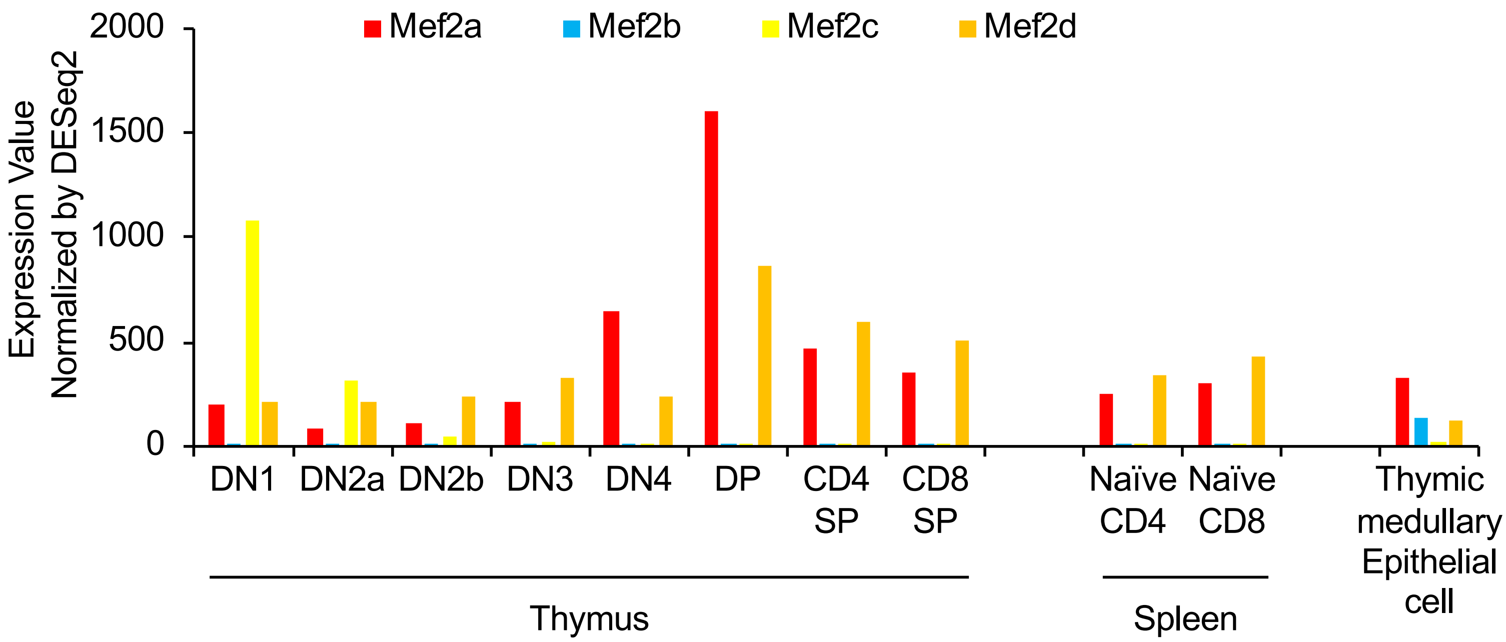

Supplementary figure 1. mRNA expression of Mef2 isoforms in T cells
Data was downloaded from the ImmGen database (www.immgen.org). The ImmGen ULI RNA Seq data set was queried using the Gene Skyline tool to obtain data for Mef2 isoform expression in thymic T cells and naïve CD4 and CD8 T cells from the spleen. Levels in thymic medullary epithelial cells are shown for comparison.
